# Controlled synthesis of poly(3-hexylthiophene) in continuous flow

**DOI:** 10.3762/bjoc.9.170

**Published:** 2013-07-25

**Authors:** Helga Seyler, Jegadesan Subbiah, David John Jones, Andrew Bruce Holmes, Wallace Wing Ho Wong

**Affiliations:** 1 School of Chemistry, Bio21 Institute, University of Melbourne, 30 Flemington Road, Parkville, Victoria 3010, Australia

**Keywords:** conjugated polymers, continuous-flow synthesis, controlled polymerization, flow chemistry, organic solar cell materials

## Abstract

There is an increasing demand for organic semiconducting materials with the emergence of organic electronic devices. In particular, large-area devices such as organic thin-film photovoltaics will require significant quantities of materials for device optimization, lifetime testing and commercialization. Sourcing large quantities of materials required for the optimization of large area devices is costly and often impossible to achieve. Continuous-flow synthesis enables straight-forward scale-up of materials compared to conventional batch reactions. In this study, poly(3-hexylthiophene), P3HT, was synthesized in a bench-top continuous-flow reactor. Precise control of the molecular weight was demonstrated for the first time in flow for conjugated polymers by accurate addition of catalyst to the monomer solution. The P3HT samples synthesized in flow showed comparable performance to commercial P3HT samples in bulk heterojunction solar cell devices.

## Introduction

**Scheme 1:**
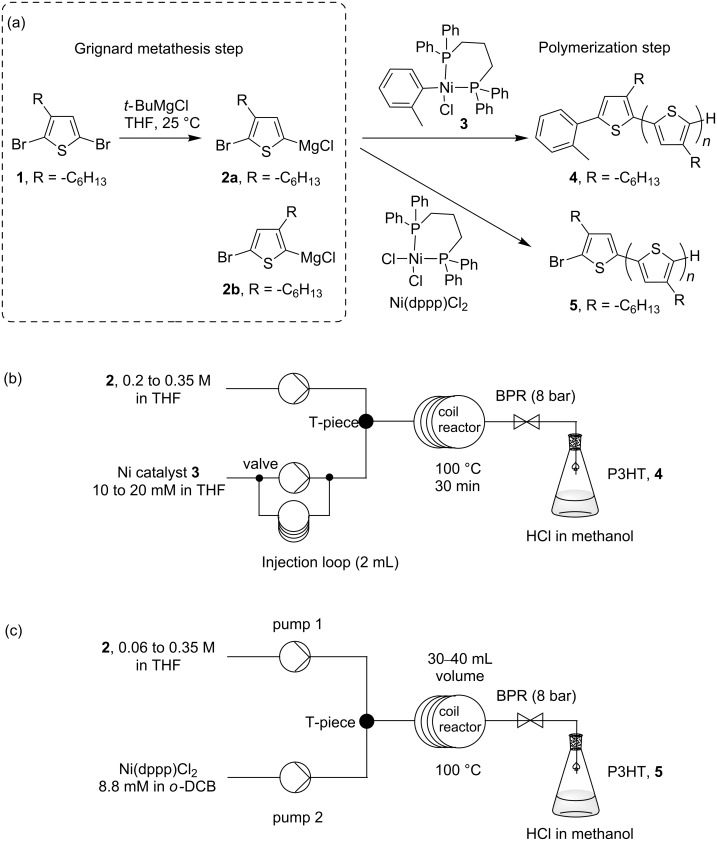
(a) Preparation of thiophene Grignard monomer and synthesis of P3HT by Kumada catalyst transfer polymerization. (b) Schematic representation for the flow setup using nickel complex **3** and (c) Ni(dppp)Cl_2_.

Poly(3-hexylthiophene), P3HT, is the most investigated material in bulk heterojunction (BHJ) organic solar cells (OSC) [1]. The reasons for its dominance in the field include its simple chemical structure, controlled synthetic method, reasonable spectral absorption, good semiconducting properties, high chemical stability, and wide commercial availability. Its emergence as a semiconducting material came with the development of synthetic methods that enable production of highly regioregular P3HT with good control of the molecular weight [2–3]. Currently, the preferred method for the synthesis of regioregular P3HT is the Kumada catalyst transfer polymerization (KCTP) or also referred to as Grignard metathesis polymerization (GRIM) [4–5]. This method is attractive because of readily available reagents, relatively mild reaction conditions, and short polymerization time. In the most basic reaction setup, 2,5-dibromo-3-hexylthiophene is treated with one equivalent of alkylmagnesium chloride to form a mixture of monosubstituted thiophene Grignard monomers (Scheme 1a). On addition of a desired amount of Ni(II) catalyst, the active catalyst species is formed in solution, and polymerization proceeds until all of the reactive Grignard monomer has been consumed. Controlled polymerization and high molecular weights have been demonstrated by many research groups with variations to this general method [6–9].

Significant quantities of materials are required for the optimization of large-area roll-to-roll printed organic solar cells [3,10]. While some of the organic semiconducting materials can be obtained commercially an a hundreds-of-grams scale, it is important to explore methods for scaled-up production in order to gain access to in-house materials at reasonable costs and lead times. In addition, batch-to-batch variations in the molecular weight distribution have been observed in commercial polymer samples, leading to differences in material deposition and film quality. This will almost certainly create problems with the performance consistency of large-area roll-to-roll printed devices. To this end, we have started to examine some key reactions in the synthesis of organic electronic materials using continuous-flow processing [11–13].

Continuous-flow synthesis methods offer several advantages over traditional batch methods [14–18]. The scale-up of reactions is not only straightforward in continuous processing, but there are also benefits in high reaction reproducibility through accurate parameter control, superior heating and mixing of reagents, boosting reaction rates, and safe handling of reactive intermediates. Using a commercial continuous-flow tube reactor [19], we have already demonstrated multigram synthesis of fullerene derivatives by cycloaddition reactions [11] as well as rapid conjugated-polymer synthesis using Suzuki–Miyaura and Stille coupling [12]. In this study, the continuous-flow synthesis of P3HT is examined. Distinct from a recent report of P3HT synthesis in a droplet-based microreactor [20], development of the flow synthesis is described in detail and controlled polymerization of P3HT, both in terms of molecular weight and regioregularity, has been achieved in this work. In particular, the feed ratio of catalyst to monomer was accurately controlled giving polymers with molecular weights ranging from 5 to 40 kg/mol as desired. In addition, BHJ devices prepared using commercial, batch and flow-synthesized P3HT gave comparable performance.

## Results and Discussion

The most widely used synthetic route to regioregular P3HT is the Kumada catalyst transfer polycondensation (KCTP) developed by the McCullough group [6] and the Yokozawa group [7]. In a representative experiment for the preparation of P3HT, the thiophene Grignard monomer **2** is prepared from the magnesium exchange reaction of an alkyl Grignard reagent and 2,5-dibromo-3-hexylthiophene (**1**, Scheme 1a). It should be noted here that a mixture of Grignard monomers **2a** and **2b** is produced in this step, typically in a ratio of 75:25. Only **2a** participates in the polymerization step on addition of the catalyst, Ni(dppp)Cl_2_, as a solid (Scheme 1a) [6]. As KCTP is a quasi-living polymerization, the product molecular weight can be controlled by adjusting the monomer-to-catalyst ratio [21].

At the start of this study, the aim was to transfer conventional batch reaction conditions for P3HT synthesis to continuous-flow processing. The polymerization step in flow was examined first with the thiophene Grignard monomer prepared in batch. Good solubility and solvent compatibility in the polymerization are essential factors to be evaluated for the translation into flow methods. The accurate addition of Ni catalyst to the thiophene Grignard monomer results in the desired catalyst-to-monomer ratio and molecular-weight control. An initial attempt was made to prepare a solution of the commonly used Ni(dppp)Cl_2_ catalyst in tetrahydrofuran (THF) which was then added to a solution of the thiophene Grignard monomer. Two problems immediately arose from this early experiment. The Ni(dppp)Cl_2_ catalyst only has modest solubility in THF at room temperature. This limited the concentration of the polymerization reaction. The second more serious problem is the dissociation of the catalyst species in THF solution. There was a visible color change in the Ni(dppp)Cl_2_/THF solution from orange to colorless over a period of several minutes. The dissociation of the catalyst species was accompanied by a decrease in catalyst activity leading to low polymer formation. To solve this problem, we were inspired by the work of a number of research groups, in which polymerization was externally initiated from an active tolyl-functionalized nickel complex **3** (Scheme 1a) [8–9,22]. The tolyl–nickel species **3** is soluble and shows good stability in THF in an inert environment. Further, polymers initiated with this complex showed lower defect levels [23–24]. After successfully experimenting with this catalyst in batch conditions, the reagent was applied in flow processing for the synthesis of P3HT (Scheme 1b).

**Figure 1:**
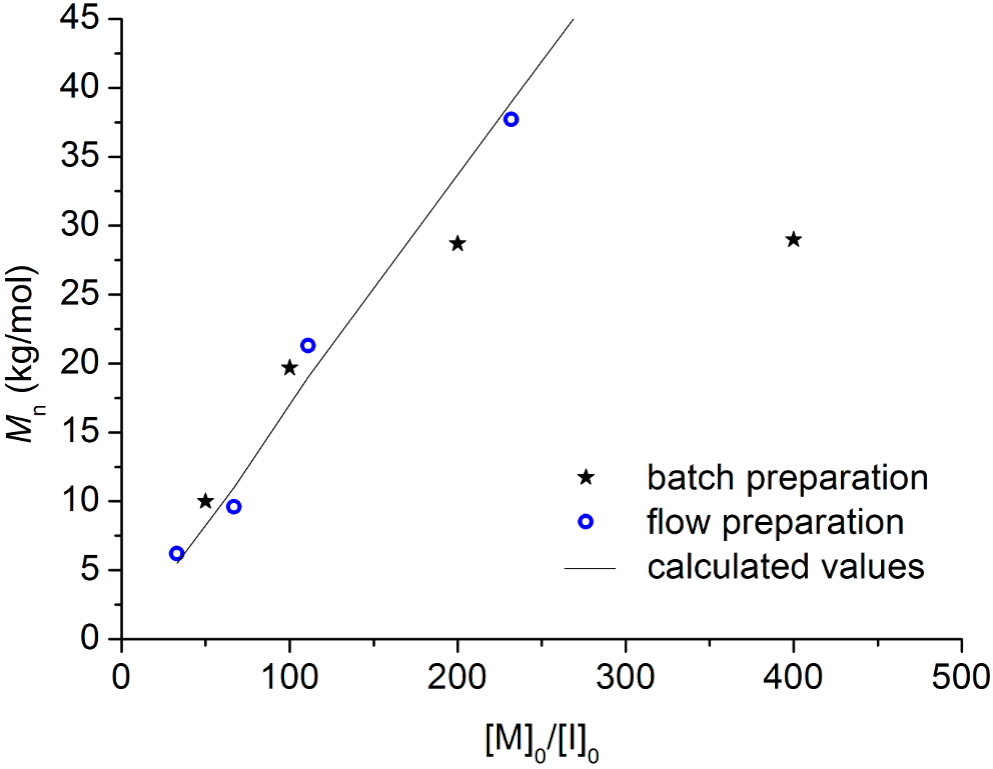
Plot of number-average molecular weight, *M*_n_, versus monomer–catalyst ratio [M]_0_/[I]_0_ for batch and flow samples prepared by external initiation using Ni catalyst **3** (see Table 1 for experimental data).

**Figure 2:**
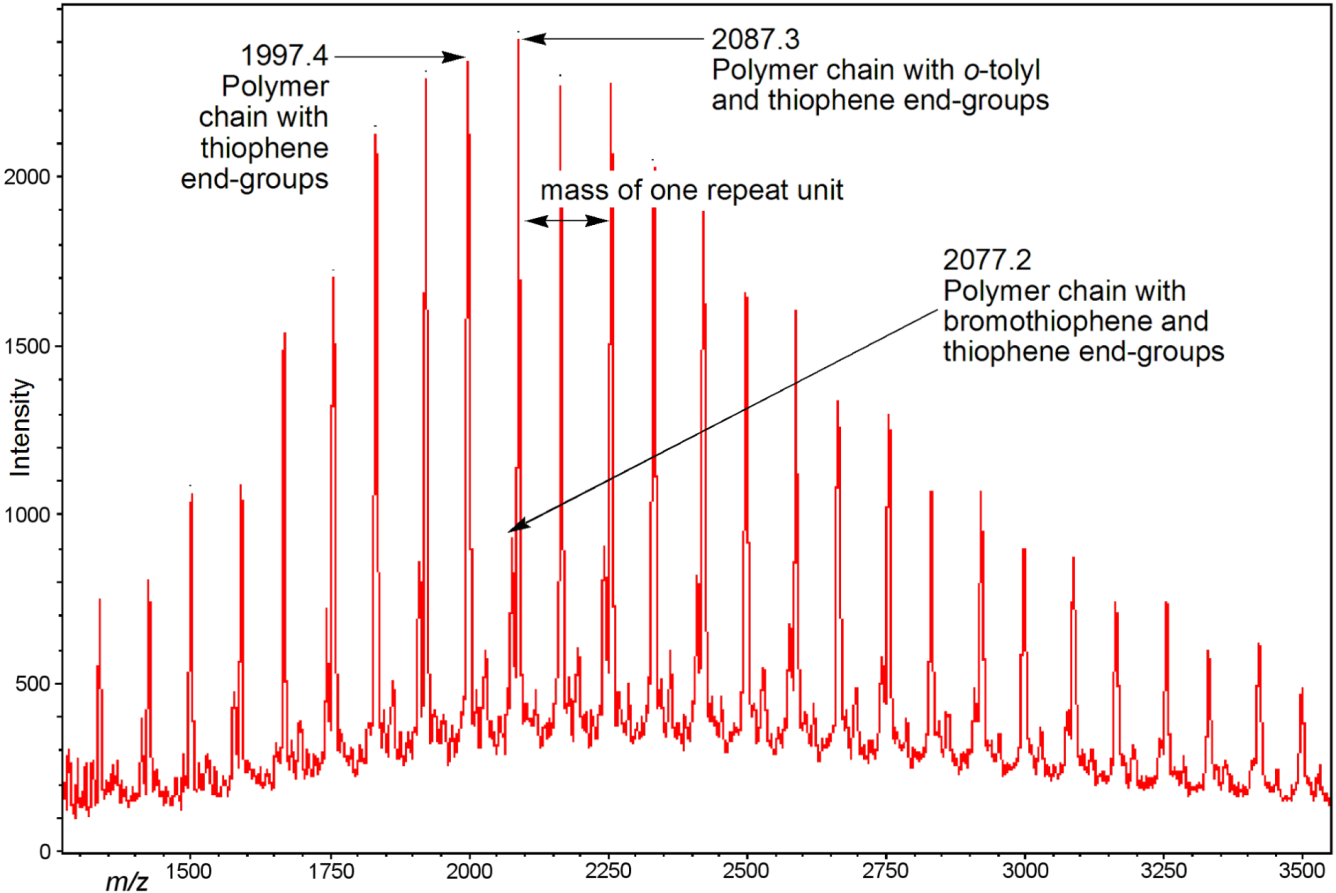
MALDI mass spectrum of low-molecular-weight preparation (GPC, *M*_n_ = 6.2 kg/mol) of P3HT in continuous flow. Signals corresponding to polymer chains with *o*-tolyl/H, H/H and Br/H end groups were observed.

The thiophene Grignard reagent **2** was prepared by traditional batch chemistry and the conversion and regioisomeric ratio were assessed in quenching experiments (Scheme 1a, ratio **2a** to **2b** 77:23; see Figure S2 in Supporting Information File 1 for ^1^H NMR data). The Ni catalyst **3** was delivered to a T-piece via an injection loop and mixed with a stream of Grignard **2** (Scheme 1b) [25]. The mixture was directed to a preheated coil reactor (polyfluoroalkoxy, PFA) at 100 °C with a retention time of 30 min. The polymer solution was then quenched in methanolic HCl (2 M) and the precipitated product was collected. The monomer-to-initiator ratio was adjusted through the variation of flow rates and reagent concentrations (Table 1). Gel-permeation chromatography (GPC) analysis in toluene (against polystyrene standards) revealed the formation of polymer with number-average molecular weights (*M*_n_) ranging from 5 to 40 kg/mol. The *M*_n_ values increased linearly with nickel catalyst content, providing evidence for the quasi-living nature [21] of the polymerization in flow processing (Table 1 and Figure 1). In fact, the molecular-weight data obtained in conventional batch reactions were very similar to those recorded in continuous-flow reactions (Figure 1). Matrix-assisted laser desorption ionization mass spectrometry (MALDIMS) and ^1^H NMR experiments were used to determine the degree of end-group control in these polymerizations [8,26]. From the mass of the polymeric species and the distinctive proton resonance of the end-groups, it was possible to detect the presence of *o*-tolyl/H, H/H and Br/H end-groups (Figure 2 and Supporting Information File 1 for details). As Ni catalyst **3** was prepared from Ni(dppp)Cl_2_ and used directly in the polymerization, the formation of different end-group species can be attributed to polymer initiation by residual Ni(dppp)Cl_2_ species [24].

**Table 1:** Flow polymerizations initiated with *o*-tolyl–nickel complex **3**.^a^

Entry	[**2**] M	Flow rate, **2** mL/min	[Catalyst **3**] M	Flow rate, **3** mL/min	Catalyst mol %	Time min	*M*_n_^b^ kg/mol	*M*_w_/*M*_n_

1	0.2	1	0.02	0.3	3	23	6.2	1.5
2	0.2	1	0.01	0.3	1.5	23	9.6	1.8
3	0.2	0.9	0.01	0.3	0.9	23	21.3	1.6
4	0.35	1^c^	0.01	0.15^c^	0.43	53	37.7^d^	1.5

^a^Polyfluoroalkoxy (PFA) tube reactor volume = 30 mL and heated at 100 °C. ^b^Molecular weight values obtained by GPC in toluene calibrated against polystyrene standards with refractive index detection. ^c^Flow rates were readjusted after the injection of reagents to afford a residence time of 53 min. ^d^Data obtained after Soxhlet extraction with methanol and petroleum spirits 40–60 °C.

While Ni catalyst **3** successfully initiated polymerizations in the time frame of this experiment, it lacked the long term stability in solution required for potential large-scale flow production of P3HT. An alternative approach was to dissolve Ni(dppp)Cl_2_ in a solvent system that limited the ligand-dissociation phenomenon observed in THF. After the screening of a number of organic solvents, Ni(dppp)Cl_2_ was found to be soluble in *o*-dichlorobenzene (*o*-DCB) and showed good stability with catalytic activity maintained over several days under normal atmospheric conditions. It is interesting to note that *o*-DCB did not adversely affect the polymerization in batch reactions despite the possibility that the aryl chloride solvent might participate in the Kumada reaction. Apparently, the reactivity of aryl chloride is significantly lower than that of aryl bromide under these reaction conditions.

**Figure 3:**
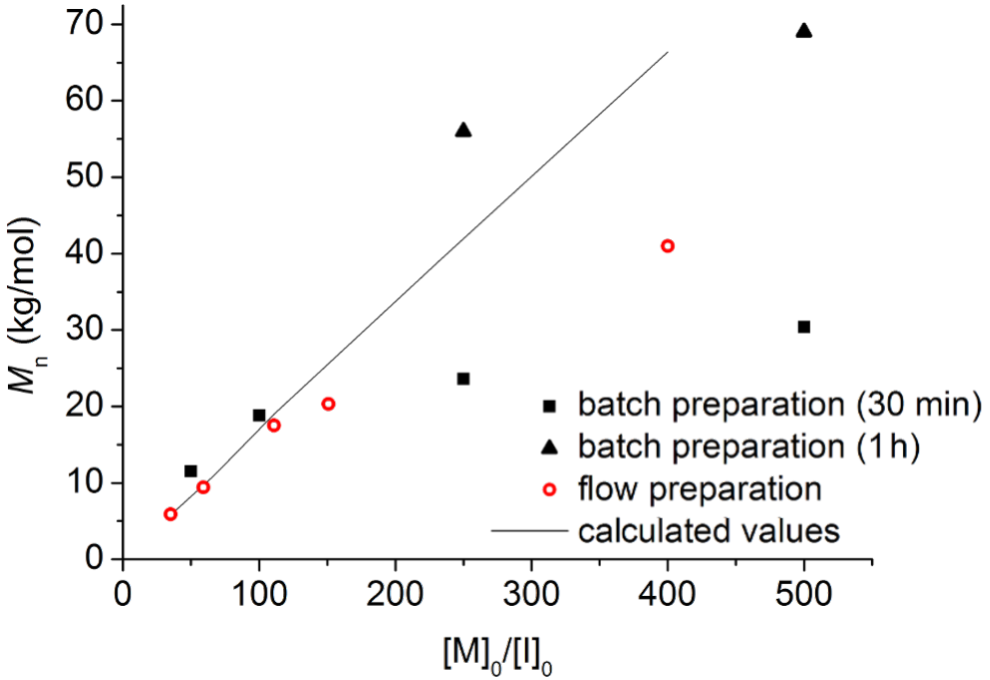
Plot of number-average molecular weight, *M*_n_, versus monomer–catalyst ratio [M]_0_/[I]_0_ for batch and flow samples prepared with Ni(dppp)Cl_2_ catalyst dissolved in *o*-DCB (see Table 2 for experimental data).

With the catalytic activity of Ni(dppp)Cl_2_ in *o*-DCB confirmed in batch reactions, continuous-flow processing was investigated (Scheme 1c, see Supporting Information File 1 for batch synthesis procedures). The preparation of various molecular weights was achieved by fine-tuning of the monomer-to-catalyst ratio [M]_0_/[I]_0_, by varying the concentration of the Grignard reagent **2** and the flow rates of the monomer and catalyst. The results for the flow polymerizations are summarized in Table 2, and Figure 3 shows the correlation between [M]_0_/[I]_0_ and molecular weight and comparison with batch experiments. The polymerization in flow compared well with the theoretical molecular weights up to 20 kg/mol. High-molecular-weight P3HT (*M*_p_ 66.7 kg/mol) was obtained in continuous flow and this was achieved in shorter reaction time compared to the batch reaction (Table 2, entry 5). The deviation from the calculated values at high molecular weights in flow processing and in batch reactions with short reaction time (30 min) indicated that longer reaction times were required (Figure 3). In batch reaction with long polymerization times (1 h), the measured molecular-weight values matched with the calculated numbers from the [M]_0_/[I]_0_ ratio (Figure 3). As our current flow setup is limited by the size of the coil reactors, we anticipate that larger coil reactor volumes would ensure high-throughput production of high-molecular-weight P3HT in continuous flow.

**Table 2:** Data for flow polymerization using Ni(dppp)Cl_2_ catalyst dissolved in *o*-DCB.^a^

Entry	[**2**] M	Flow rate, **2** mL/min	Flow rate, Ni(dppp)Cl_2_ mL/min	Catalyst mol %	Time min	*M*_n_ (*M*_calcd_) kg/mol	*M*_p_ kg/mol	*M*_w_/*M*_n_

1	0.06	1	0.2	2.9	33	5.9 (5.6)	13	2
2	0.2	1	0.4	1.7	30	9.6 (9.4)	22	1.9
3	0.2	0.9	0.18	0.9	27	17.5 (18)	31	1.6
4	0.35	0.7	0.18	0.7	34	20.3 (25.1)	33.5	1.5
5	0.35	1^b^	0.1^b^	0.25	50	41^c^ (66)	66.7^c^	1.4

^a^Stainless-steel tube reactor volume = 40 mL for entries 1 and 2; PFA tube reactor volume = 30 mL for entries 3 to 5. Reactors heated to 100 °C. ^b^Flow rate was readjusted after injection of reagents to afford 50 min residence time. ^c^After Soxhlet extraction with methanol and petroleum spirits 40–60 °C.

In our studies, stainless-steel tube reactors were also examined for the synthesis of P3HT. In comparison to polyfluoroalkoxy (PFA) tube reactors, stainless-steel tube reactors offer the advantage of low gas permeability. Satisfactory results were achieved for relatively low degrees of polymerization in the stainless-steel reactors (Table 2, entries 1 and 2). Interestingly, higher molecular weights could only be achieved in the PFA reactors (Table 2, entries 3–5). This suggests that the nickel-catalyzed polymerization is incompatible with the stainless-steel reactor and we speculate that the nickel content in stainless steel may be the cause of the incompatibility especially at the elevated reaction temperatures used.

**Scheme 2:**
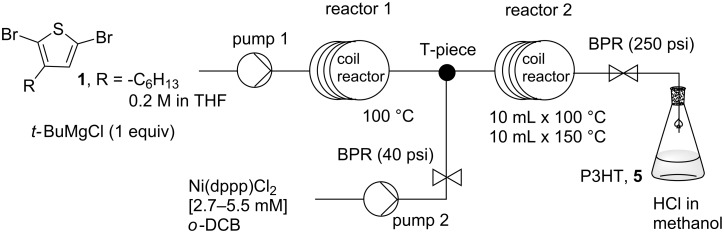
Schematic representation of the telescoped preparation of P3HT in a flow reactor.

To demonstrate the flow synthesis of P3HT from 2,5-dibromo-3-hexylthiophene (**1**), the Grignard metathesis step was performed in the tube reactor followed by the addition of the Ni(dppp)Cl_2_ catalyst for the polymerization (Scheme 2). In this telescoped process, the thiophene Grignard compounds **2a** and **2b** were formed in the first coil and subsequently mixed with the catalyst stream and fed into the second reactor (Scheme 2). The two-step reaction was performed under superheated conditions (at 250 psi back pressure) and the reagent flow rates were adjusted to deliver reasonable reaction times for both reactors. For the Grignard metathesis step, a stock solution containing both 2,5-dibromo-3-hexylthiophene (**1**) and the *tert*-butylmagnesium chloride (0.2 M in THF) were allowed to react at 100 °C with a retention time of 20 min in reactor coil 1.

**Figure 4:**
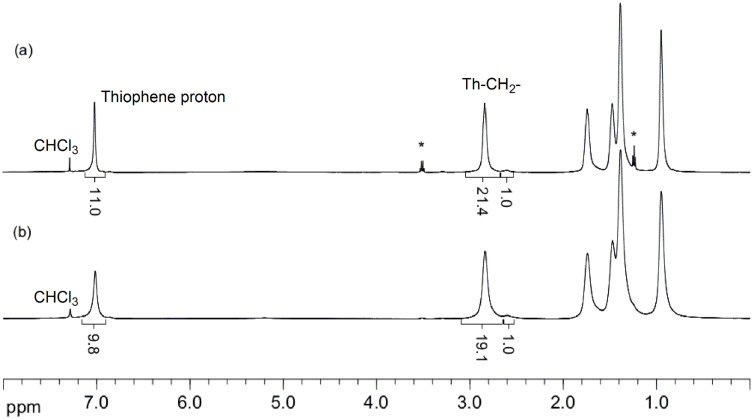
^1^H NMR (CDCl_3_, 500 MHz) spectra of P3HT samples prepared in (a) flow and (b) batch show comparable regioregularity of approximately 95% for both processes. P3HT prepared in flow (*M*_n_ = 20 kg/mol) and in batch (*M*_n_ = 17 kg/mol) (* designates residual solvent).

Ni(dppp)Cl_2_ catalyst in *o*-DCB was added to this reaction stream and the polymerization was heated at 150 °C for 18 min. Under these conditions, we succeeded in obtaining P3HT with *M*_n_ of 31 kg/mol and polydispersity of 1.5 (Table 3, entry 1). The regioregularity was estimated to be 95% from the integration of the α-methylene protons in the ^1^H NMR spectrum (Figure 4). The same degree of regioregularity was observed for a batch sample of similar molecular weight. Additionally, the variation of the molecular weight was investigated when the [M]_0_/[I]_0_ ratio was varied. As expected, increasing the concentration or the flow rate of the catalyst stock solution, afforded lower-molecular-weight polymers (Table 3, entries 2 and 3).

**Table 3:** Data for the telescoped synthesis of P3HT from 2,5-dibromo-3-hexylthiophene (**1**) in flow.^a^

Entry	Flow rate, monomer mL/min	[Catalyst] mM	Flow rate, Ni(dppp)Cl_2_ mL/min	Catalyst mol %	Time^b^ min	*M*_n_ kg/mol	*M*_p_ kg/mol	*M*_w_/*M*_n_

1	1	2.7	0.1	0.14	18	31^c^	49^c^	1.5^c^
2	1	5.5	0.1	0.28	18	20.5	47	1.9
3	1	5.5	0.18	0.5	28^d^	8	16	1.8

^a^Reactor 1 (PFA × 2) volume = 20 mL; reactor 2 (PFA and stainless steel) = 20 mL. ^b^Residence time for the polymerization. ^c^After Soxhlet extraction with methanol and petroleum spirits 40–60 °C. ^d^Reaction time was adjusted by slowing down the flow rate after reactor 1.

**Figure 5:**
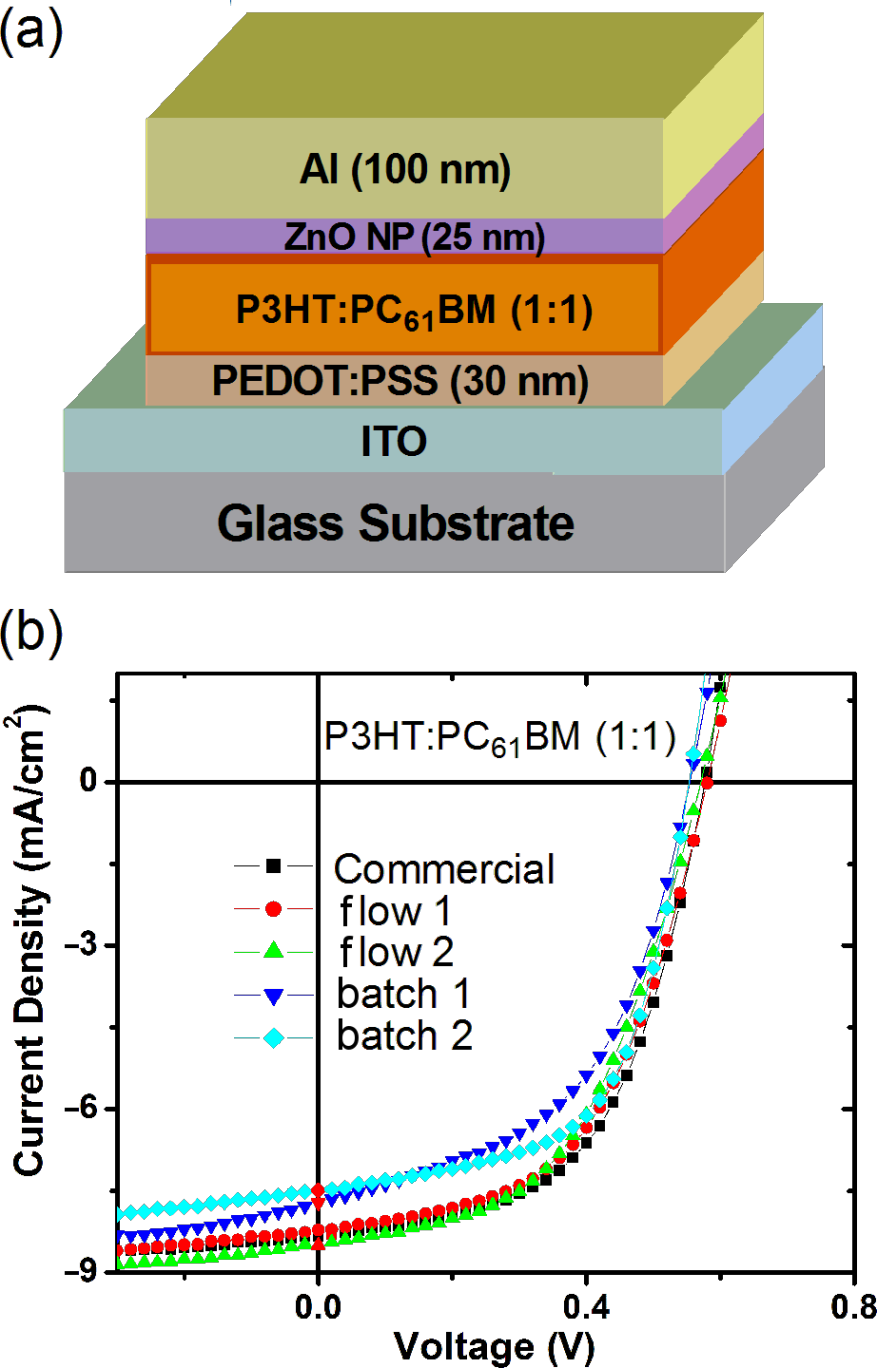
(a) Schematic diagram of the photovoltaic device geometry and (b) *J*–*V* curves of BHJ solar cells with P3HT:PC_61_BM active layer deposited by spin coating from chlorobenzene solution. Commercial = Merck P3HT (lisicon SP001); flow 1 = Table 1, entry 4; flow 2 = Table 2, entry 5; batch 1 = P3HT synthesized with catalyst **3**; batch 2 = P3HT synthesized with Ni(dppp)Cl_2_.

In order to assess the device performance of P3HT synthesized using flow methods described in this study, bulk heterojunction (BHJ) solar cells were fabricated and tested. BHJ devices were fabricated with the following device geometry: ITO/PEDOT:PSS/P3HT:PC_61_BM (1:1)/ZnO nanoparticle/Al, where ITO = indium tin oxide, PEDOT:PSS = poly(3,4-ethylenedioxy-thiophene):poly(styrenesulfonate) and PC_61_BM = [6,6]-phenyl-C_61_-butyric acid methyl ester (Figure 5a). The P3HT:PC_61_BM active layer was deposited by spin coating from chlorobenzene solution. Device fabrication and characterizations were performed in air (see Supporting Information File 1 for details).

The current-density–voltage (*J*–*V*) curves for the solar-cell devices prepared from various P3HT samples are shown in Figure 5b. The device performance parameters for the different P3HT samples were all rather similar with power conversion efficiency (PCE) ranging from 2.1 to 2.6% (Table 4). The short circuit current density (*J*_sc_) parameter showed the largest variations and this directly correlated with the thickness of the device active layer (Table 4). Thinner active layers absorbed less of the incoming irradiation leading to decreased *J*_sc_. It is pleasing to note that the performance of the commercial P3HT sample (Merck lisicon SP001) was comparable to that of P3HT from flow synthesis (Table 4). It should be noted that the lower PCE of these devices compared with some literature values [1] was attributed to the fact that all devices were fabricated and measured in air. Interestingly, P3HT samples with different end-group variations did not have significant effects on device performance. These results are extremely encouraging for the product of device-grade P3HT using continuous-flow methods with simple benchtop equipment.

**Table 4:** Solar cell data for devices containing various P3HT samples.^a^

P3HT^b^	*M*_n_ kg/mol	Thickness^c^ nm	*J*_sc_ mA/cm^2^	*V*_oc_ V	FF %	PCE %

Merck	50	145	8.30	0.58	54	2.60
Flow 1	38	160	8.20	0.58	53	2.54
Flow 2	41	175	8.50	0.58	50	2.47
Batch 1	24	125	7.50	0.56	58	2.45
Batch 2	34	140	7.70	0.56	50	2.15

^a^*J*_sc_ = short circuit current density; *V*_oc_ = open circuit voltage; FF = fill factor; PCE = power conversion efficiency; performance shown was the average from 6 pixels with an area of 0.1 cm^2^. ^b^Merck = Merck lisicon SP001; flow 1 = Table 1, entry 4; flow 2 = Table 2, entry 5; batch 1 = P3HT synthesized with catalyst **3**; batch 2 = P3HT synthesized with Ni(dppp)Cl_2_. ^c^Thickness of active layer. Measurements were performed in air.

## Conclusion

P3HT has been successfully synthesized in continuous flow by using a commercially available benchtop flow reactor. After optimization of reaction conditions, good molecular-weight control was achieved by adjusting the monomer-to-catalyst ratio with variations in reagent concentration and flow rates. This methodology enables the controlled synthesis of conjugated polymers in flow exclusively by the adjustment of the feed of monomer and initiator into the tube reactor. The major challenge in this study was to find compatible nickel catalyst systems for the Kumada polymerization step in flow. Both *o*-tolyl–Ni catalyst **3** in THF and Ni(dppp)Cl_2_ in *o*-DCB were highly active in continuous processing conditions, and high-molecular-weight P3HT was obtained for both catalysts. Apart from the molecular weight of the polymers, the P3HT samples synthesized in flow showed comparable regioregularity to samples from batch synthesis. The telescoped flow synthesis of P3HT from 2,5-dibromo-3-hexylthiophene (**1**) was also achieved on the benchtop flow reactor. Finally, BHJ solar cells containing flow-synthesized P3HT showed comparable performance to both commercial and batch-synthesized samples. This study paves the way for the continuous-flow synthesis of other polymers using the KCTP method. With efficient synthesis of polymers possible in a benchtop flow reactor, scale-up of such materials will become straight forward leading to reduction in production costs and improvements in reproducibility.

## Experimental

General: The continuous-flow experiments were conducted by using a Vapourtec R2/R4 unit with multiple perfluoroalkoxy PFA (10 mL internal volume) or stainless steel (10 mL internal volume) reactors connected in series. All solvents were degassed and reactions were performed under anaerobic conditions. The reactants were channelled into the tube reactor by pumping solvent from a reservoir. Residence times in the reactor coils were defined by the flow rate and the volume of the reactor. The system was thoroughly dried by first flushing with anhydrous methanol followed by dried acetone before refilling with anhydrous reaction solvent. Reported yields do not account for axial dispersion of the reaction mixture and the formation of the regioisomer **2b**. Gel permeation chromatography (GPC) data was obtained by using toluene as the eluent and calibrated against polystyrene reference standards.

Materials: 2,5-Dibromo-3-hexylthiophene [27], 2-bromo-5-chloromagnesium-3-hexylthiophene [28], and *cis*-chloro(2-tolyl)(dppp)nickel(II) complex [8] were prepared according to procedures described in the literature. Commercial reagents were used as purchased without further purification. Commercial P3HT (lisicon SP001, *M*_n_ 50 kg/mol, *M*_w_ 79 kg/mol) was acquired from Merck KGaA, Darmstadt, Germany.

Synthetic procedure for 2-bromo-5-chloromagnesium-3-hexylthiophene (**2**): *tert*-Butylmagnesium chloride (8 mL) was added dropwise to a solution of 2,5-dibromo-3-hexylthiophene (1.826 g, 5.6 mmol) in dry THF (20 mL) and the mixture was stirred overnight at room temperature. An aliquot was quenched in water and extracted with petroleum spirits 40–60 °C, and the conversion was monitored by ^1^H NMR spectroscopy.

Flow synthesis of P3HT via external initiation with nickel catalyst **3**: Stock solutions containing 2-bromo-5-chloromagnesium-3-hexylthiophene (**2**) (0.35/0.2 M) and Ni(dppp)(*o*-tolyl)Cl **3** (20/10 mM) were pumped into the PFA coil reactor (3 × 10 mL, 100 °C) at specific flow rates (see Table 1). The variation of the respective concentrations and flow rates afforded four different monomer-to-initiator ratios (3, 1.5, 0.9, 0.43 mol %). The stream outlet was fitted with an 8 bar back-pressure regulator. The mixtures were quenched and precipitated from methanolic HCl (2 M), centrifugated and washed with the same solvent three times. The lower molecular weight sample was then washed with acetone, and the high-molecular-weight samples were washed with petroleum spirits 40–60 °C. Yield (*M*_n_ = 37.7 kg/mol): 37 mg, 43%. ^1^H NMR (CDCl_3_, 500 MHz) δ 0.92 (t, 3H, *J* = 7 Hz), 1.32–1.37 (m, 4H), 1.41–1.46 (m, 2H), 1.68–1.74 (m, 2H), 2.49 (s, 0.06), 2.81 (t, 2H, *J* = 7.7 Hz), 6.98 (s, 1H), 7.16 (d, 0.02H, *J* = 5.2 Hz), 7.22–7.25 (m, 0.03H), 7.43–7.45 (m, 0.02H); ^13^C NMR (CDCl_3_, 100 MHz) δ 139.87, 133.69, 130.47, 128.58, 31.69, 30.50, 29.46, 29.26, 22.65, 14.12.

Flow synthesis of P3HT by in situ initiation with Ni(dppp)Cl_2_: Stock solutions (0.35 M/0.2 M/0.06 M) containing 2-bromo-5-chloromagnesium-3-hexylthiophene (**2**) and Ni(dppp)Cl_2_ (8.8 mM) were pumped into the PFA coil reactor (3 × 10 mL, 100 °C, PFA) or into the stainless steel (SS) reactor (40 mL, 100 °C) at specific flow rates (see Table 2). The variation of the concentration of the thiophene monomer and adjusting of the flow rates afforded five different monomer-to-initiator ratios (2.9, 1.7, 0.9, 0.7, 0.25 catalyst mol %). The stream outlet was fitted with an 8 bar back-pressure regulator. The reaction mixtures were quenched by direct precipitation of the stream leaving the reactor, from methanolic HCl (2 M). The polymers were centrifugated and washed with the same solvent three times. The lower molecular weight sample was then washed with acetone, whereas the rest of the samples were washed with petroleum spirits 40–60 °C. Yield (*M*_n_ 5.9 kg/mol): 32 mg, 65%.^1^H NMR (CDCl_3_, 500 MHz) δ 0.91 (m, 3H), 1.25–1.44 (m, 6H), 1.70 (m, 2H), 2.82 (s, 2H), 6.98 (s, 1H); ^13^C NMR (CDCl_3_, 100 MHz) δ 139.88, 133.69, 130.47, 128.59, 31.69, 30.50, 29.46, 29.25, 22.65, 14.11.

Telescoped preparation of P3HT: A stock solution containing 2,5-dibromo-3-hexylthiophene (0.2 M, THF) and *tert*-butylmagnesium chloride (1 equiv) was pumped into the PFA coil reactor (2 × 10 mL, 100 °C) at 1 mL/min flow rate, resulting in 20 min residence time. The solution was then mixed with a second stream containing Ni(dppp)Cl_2_ catalyst (2.7 mM) at 0.1 mL/min to give a 0.1 mol % catalyst content and directed into a series of two reactors (at 100 °C or/and 150 °C). The inner pressure of the system was adjusted to give a continuous steady flow using a 250 psi back-pressure regulator located at the outlet of the system. The mixture was quenched into methanol, centrifuged and washed with the same solvent three times. The concentrations of the catalyst and flow rates were varied to adjust the monomer-to-initiator ratio (see Table 3). The polymer was subjected to Soxhlet extraction with methanol and petroleum spirits (40–60 °C). Yield (*M*_n_ = 31 kg/mol): 70 mg, 40%. ^1^H NMR (CDCl_3_, 500 MHz) δ 0.94 (s, 3H), 1.23–1.55 (m, 6H), 1.73 (m, 2H), 2.82 (s, 2H), 7.0 (s, 1H); ^13^C NMR (CDCl_3_, 100 MHz) δ 139.88, 133.71, 130.49, 128.59, 31.70, 30.51, 29.26, 22.65, 14.11.

## Supporting Information

File 1 Synthetic procedures for batch reactions, characterization of P3HT samples including NMR and MALDI-TOF spectra, and procedures for device preparation.
