# Supplementary material for: Controlled synthesis of poly(3-hexylthiophene) in continuous flow
Source: Beilstein J Org Chem. 2013 Jul 25;9:1492–500. doi: 10.3762/bjoc.9.170 (PMC3740766; doi:10.3762/bjoc.9.170)
Supplement: File 1 — Synthetic procedures for batch reactions, characterization of P3HT samples including NMR and MALDI-TOF spectra, and procedures for device preparation. [file Beilstein_J_Org_Chem-09-1492-s001.pdf]

Supporting Information

for

Controlled synthesis of poly(3-hexylthiophene) in continuous flow

Helga Seyler, Jegadesan Subbiah, David J. Jones, Andrew B. Holmes and Wallace W. H. Wong*

Address: School of Chemistry, Bio21 Institute, University of Melbourne, 30 Flemington Road, Parkville, Victoria 3010, Australia

Email: Wallace W. H. Wong - wwhwong@unimelb.edu.au

*Corresponding author

Synthetic procedures for batch reactions, characterization of P3HT samples including NMR and MALDI-TOF spectra, and procedures for device preparation

Contents:

General experimental S2
Synthetic procedures S3
NMR and MALDI data S5
Solar cell devices S8
References S9

General experimental setup

Instruments

The continuous-flow experiments were conducted using a Vapourtec R2/R4 unit (http://www.vapourtec.co.uk/) with multiple perfluoroalkoxy PFA (10 mL internal volume) or stainless steel (10 mL internal volume) reactors connected in series. All solvents were degassed and reactions were performed under anaerobic conditions. The reactants were channelled into the tube reactor by pumping solvent from a reservoir. Residence times in the reactor coils were defined by the flow rate and the volume of the reactor. The system was thoroughly dried by first flushing with anhydrous methanol followed by dried acetone before refilling with anhydrous reaction solvent.

^{1}H and ^{13}C NMR measurements were carried out from $CDCl_3$ solutions on either a 500 MHz or a 400 MHz instrument. Gel permeation chromatography (GPC) data was obtained using a Viscotek GPC Max VE2001 solvent/sample module equipped with a Viscotek VE3580 refractive index detector. Toluene was used as the eluent with a 200 µL sample volume injection. Samples were passed through three 30 cm, PL gel (5 µm) mixed C columns and one 30 cm, PL gel (3 µm) mixed E column at 0.6 mL/min. Molecular mass distributions were calculated relative to narrow polystyrene reference standards.

Materials

2,5-Dibromo-3-hexylpthiophene [1], 2-bromo-5-chloromagnesium-3-hexylthiophene [2] and *cis*-chloro(2-tolyl)(dppp)nickel(II) complex [3] were prepared according to procedures described in the literature. Commercial reagents were used as purchased without further purification. Commercial P3HT (lisicon SP001, M_n 50 kg/mol, M_w 79 kg/mol) was acquired from Merck KGaA, Darmstadt, Germany.

Synthetic procedures

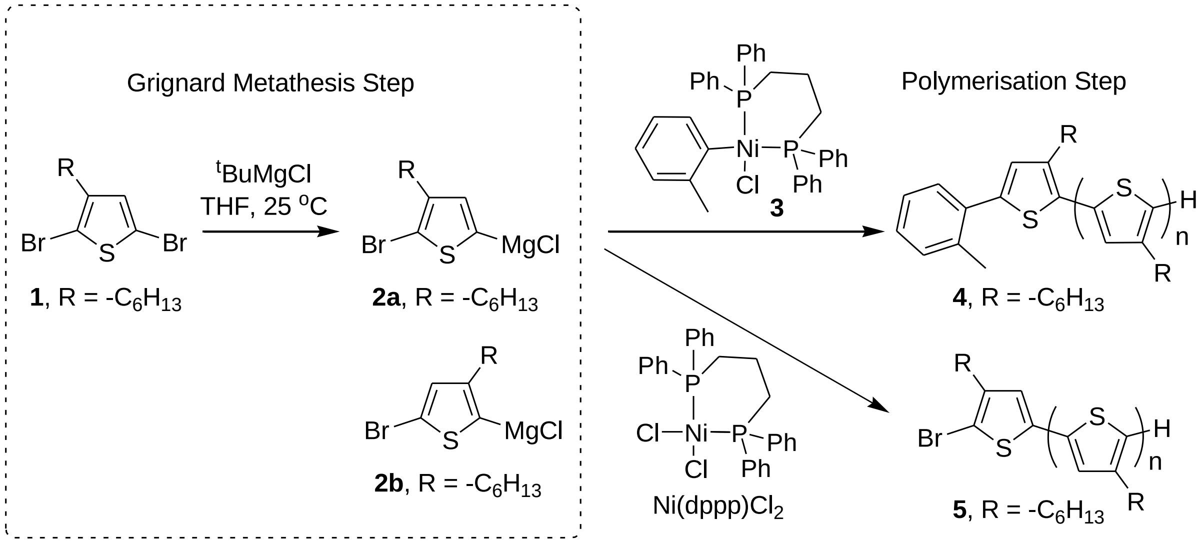

Scheme S1. Synthesis of P3HT by Kumada catalyst transfer polymerisation.

KCTP for the preparation of P3HT by external initiation with Ni(dppp)Cl_2

Batch procedure: A Radleys carousel was loaded with five reactor tubes containing 2,5-dibromo-3-hexylthiophene (0.710 mg, 2.18 mmol), *tert*-butylmagnesium chloride (2.4 mmol) in anhydrous THF. The reactors were heated under reflux for 1 h, followed by the addition of Ni(dppp)Cl_2 in *o*-DCB (8.7 mM, 5 mL, 2.5 mL, 1 mL, 0.5 mL). Aliquots were taken after 30 min and 1 h. After heating the mixture for 2 h under reflux, the reaction was quenched with 2 M methanolic HCl, and the purple precipitate was collected and washed with methanol.

Table S1. Batch polymerisations initiated with Ni(dppp)Cl_2 in *o*-DCB.

Entry	Catalyst [mol %]	M_n [$M_{n,theo}$] [kg/mol]	PDI
1	2	11.5 [8.3]	1.3
2	1	19 [16.6]	1.4
3	0.4	23.6 [41.5]	1.6
4	0.2	30.4 [83]	1.7

KCTP for the preparation of P3HT via in situ initiation with nickel catalyst **3**

Batch procedure: To a solution of 2-bromo-5-chloromagnesium-3-hexylthiophene **2** (5 mL, [0.2 M] in THF) was added *cis*-chloro(2-tolyl)(dppp)nickel(II) complex ([0.031 M] in THF; 0.64 mL, 0.32 mL, 0.16 mL, 80 μL) to afford four polymers with 2, 1, 0.5, 0.25 catalyst mol % content, respectively. The reaction was quenched after 1 h under reflux. The final polymer was precipitated in methanolic HCl (2 M), washed with MeOH and petroleum spirits 40–60 °C. Yield: 107 mg (64%, 10 kg/mol). ^{1}H NMR ($CDCl_3$, 500 MHz) δ 0.92 (t, 3H, J = 7 Hz), 1.34-138 (m, 4H), 1.41-1.46 (m, 2H), 1.69-1.74 (m, 2H), 2.49 (s, 0.1 H), 2.82 (t, 2H, J = 7.7 Hz), 6.98 (s, H), 7.16 (d, 0.03H, J = 5.2 Hz), 7.22-7.24 (m, 0.06H), 7.42-7.44 (m, 0.03H).^{13}C NMR ($CDCl_3$, 100 MHz) δ 139.88, 133.69, 130.47, 128.59, 31.69, 30.50, 29.46, 29.26, 22.65, 14.12.

Table S2. Batch polymerisations initiated with *cis*-chloro(2-tolyl)(dppp)nickel(II) **3**.

Entry	Catalyst [mol %]	M_n [$M_{n,theo}$] [kg/mol]	M_p [kg/mol]	PDI
1	2	10 [8.3]	16.8	1.5
2	1	19.7 [17]	35	1.4
3	0.5	28.7 [33]	55	1.4
4	0.2	29 [66]	63.7	1.6

NMR and MALDIMS data

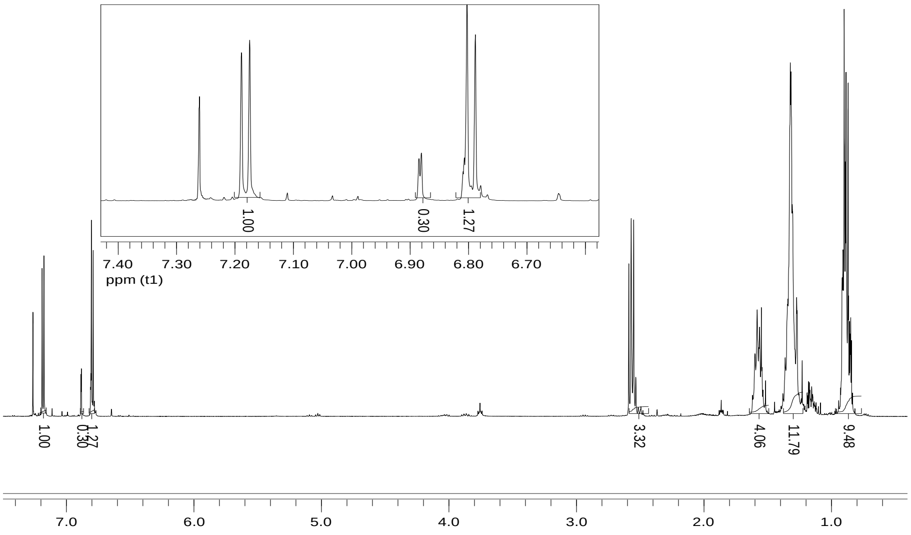

Figure S1. ^{1}H NMR ($CDCl_3$, 400 MHz) spectrum of the quenched monomer after the magnesium-halogen exchange reaction in batch (regioisomeric mixture 77:23 of compounds **2a** and **2b** upon quenching with methanolic HCl).

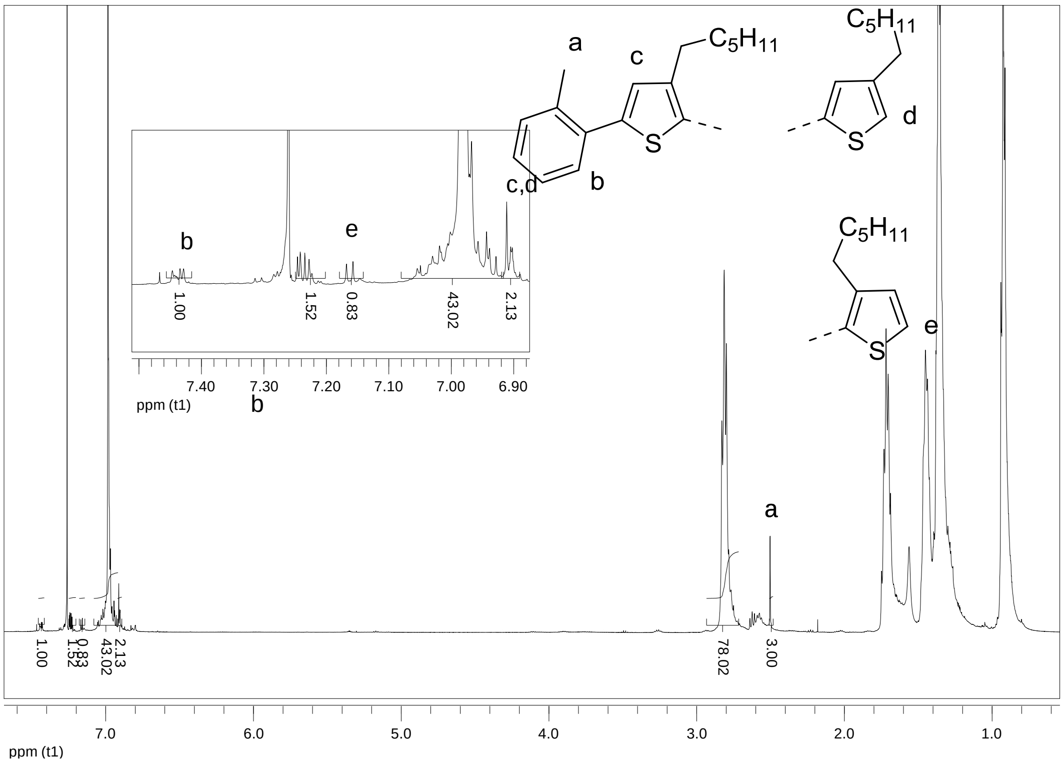

Figure S2. ^{1}H NMR spectrum ($CDCl_3$, 500 MHz) and expanded section (inset) of a low-molecular-weight P3HT sample prepared in flow by external initiation.

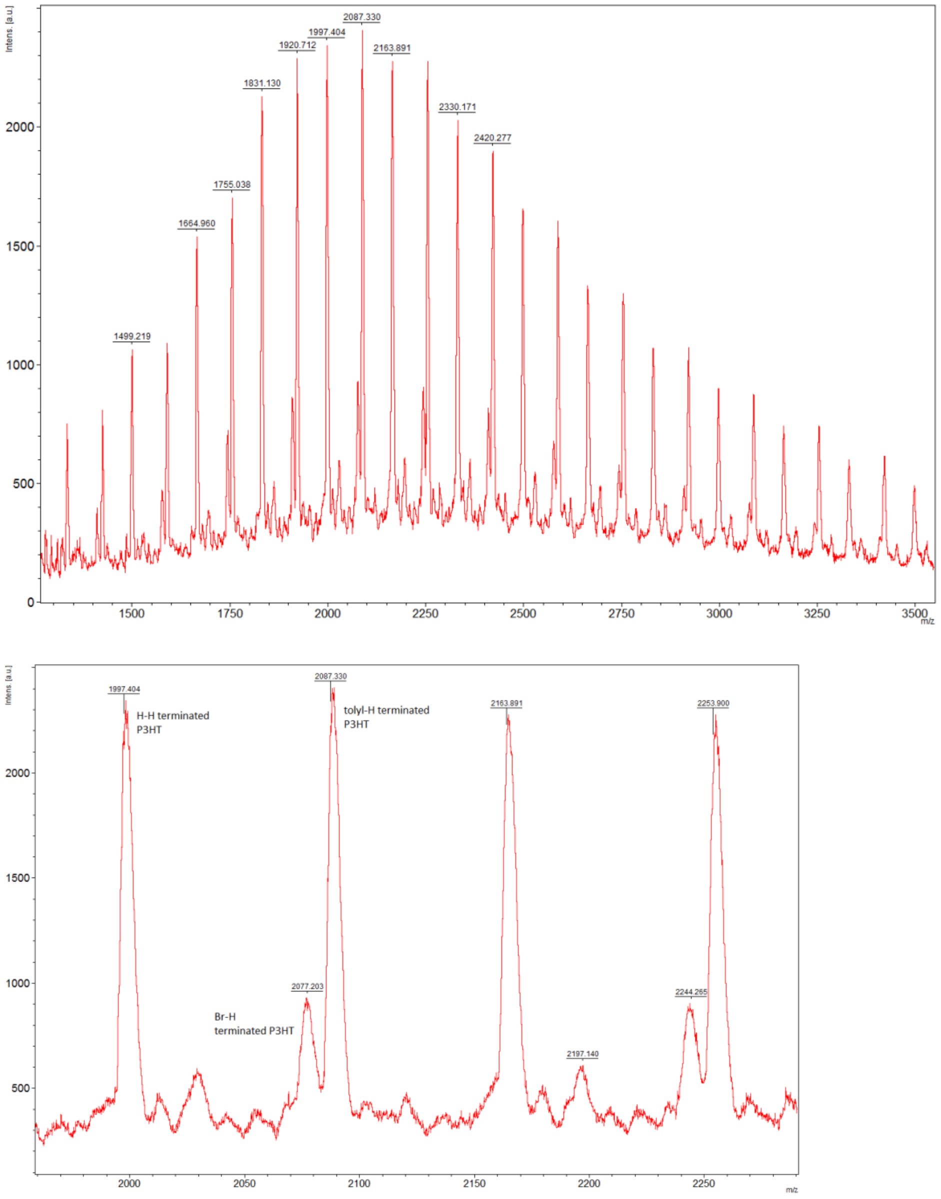

Figure S3. MALDI-TOF spectrum (top) and expanded section (bottom) of a low-molecular-weight P3HT sample prepared in flow by external initiation. H/tolyl and H/H and H/Br terminated chains were detected.

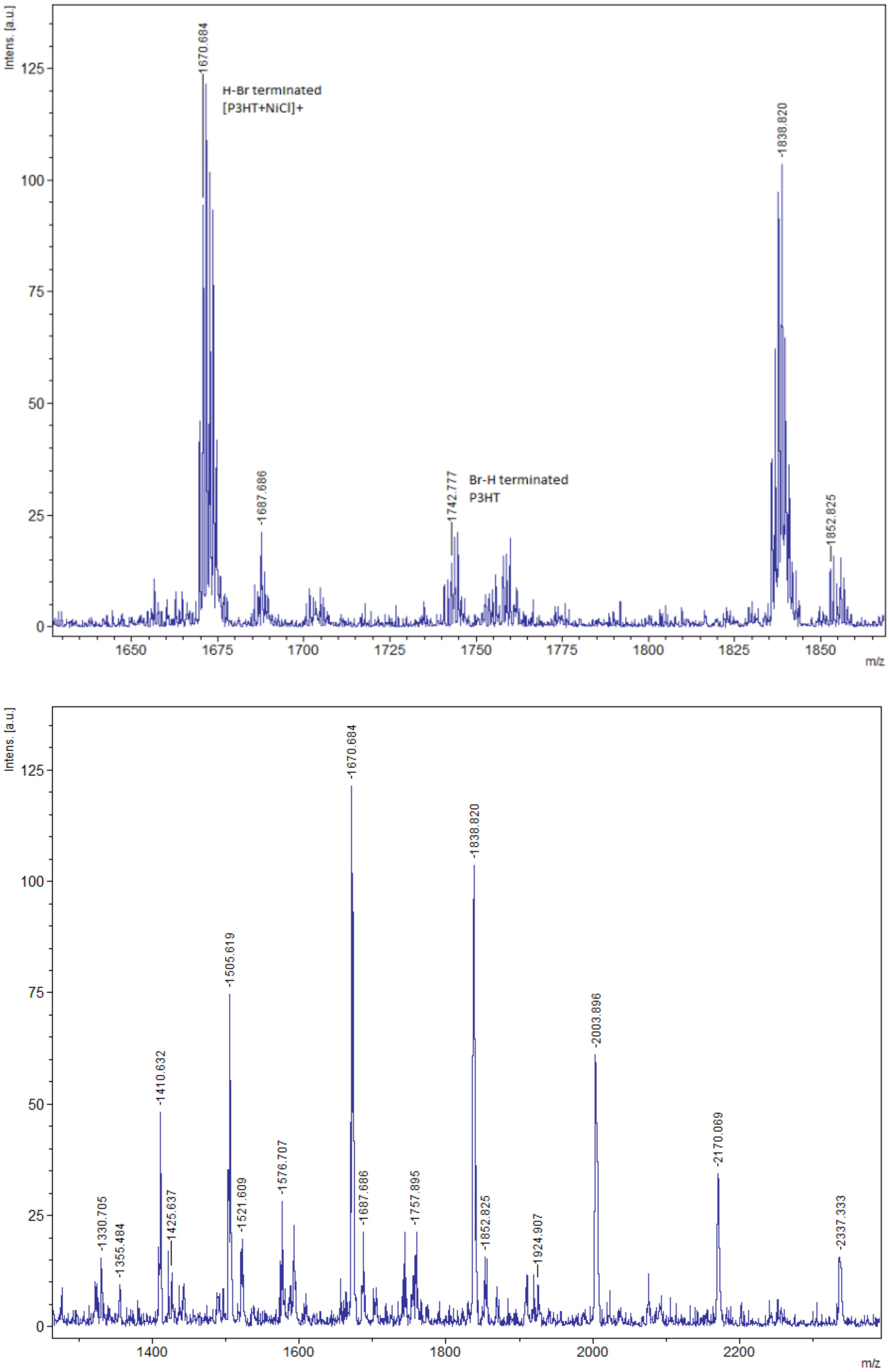

Figure S4. MALDI-TOF spectrum (top) and expanded section (bottom) of a low-molecular-weight P3HT sample prepared in flow by in situ initiation.

Bulk heterojunction solar cell devices

Device fabrication

Polymer solar cells were processed on pre-patterned indium tin oxide (ITO)-coated glass substrates with a sheet resistance of 15 Ω per square. First a thin layer (30 nm) of poly(3,4-ethylenedioxythiophene):poly(styrenesulfonate) (PEDOT:PSS; Baytron AI 4083 from HC Starck) was spin-coated on a ultrasonically cleaned ITO substrate, followed by baking on a hot plate at 140 °C for 10 min. An active layer of the device was deposited by spin coating a chlorobenzene solution containing 12 mg of P3HT and 12 mg of $PC_{60}BM$. A thin layer of ZnO nanoparticles was deposited on the active layer by spin coating (3000 rpm) to form 25 nm of ZnO layer [4] followed by annealing on a hot plate at 150 °C for 2 min. The films were then transferred to a metal evaporation chamber and aluminum (100 nm) was deposited through a shadow mask (active area was 0.1 cm^2) at approximately 1 x 10^{-6} torr. Film thickness was determined by Veeco Dektak 150+Surface Profiler. The current-density–voltage measurements of the devices were carried out using a 1 kW Oriel solar simulator with an AM 1.5G filter as the light source in conjunction with a Keithley 2400 source measurement unit. Solar measurements were carried out under 1000 W/m^2 AM 1.5G illumination conditions. For accurate measurement, the light intensity was calibrated using a reference silicon solar cell (PVmeasurements Inc.) certified by the National Renewable Energy Laboratory. Device fabrication and characterizations were performed in an ambient environment without any encapsulation.

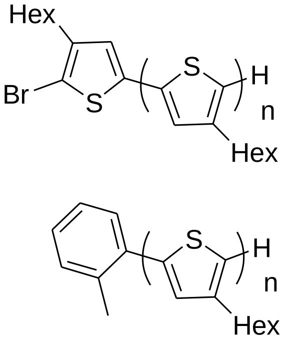

B-34Th
F-41Th

B-24Tol
F-38Tol

Entry	P3HT	Synthetic method	M_n [kg/mol]	M_w [kg/mol]
1	B-34Th	batch	34	54
2	F-41Th	flow	41	58
3	B-24Tol	batch	24	36
4	F-38Tol	flow	38	57

Figure S5. P3HT prepared in flow and batch reactions. M_n, M_w and PDI obtained after Soxhlet extraction with MeOH and petroleum spirits 40–60 °C.

Process Conditions:

Soln: P3HT:PC60BM (1:1)

Chlorobenzene (24 mg/ml)

Spin coating 1200 rpm

Annealing 150°C

Device Geometry:

ITO/PEDOT:PSS/P3HT:PCBM/ZnO/Al

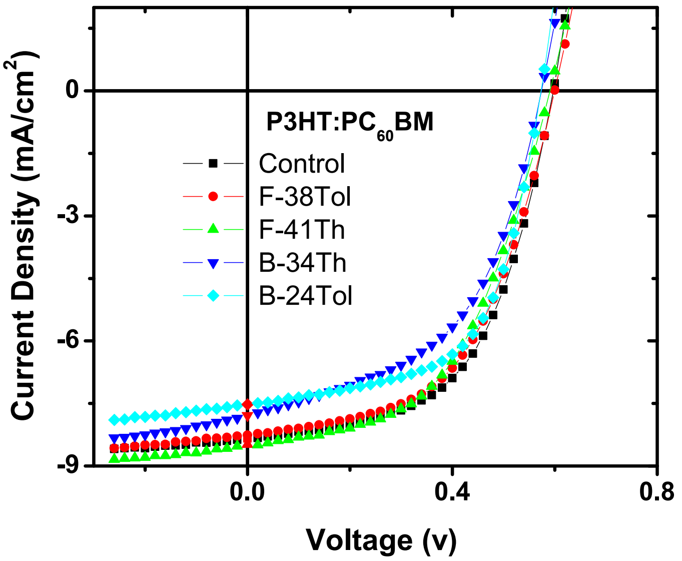

Figure S6. Solar cell device geometry and process conditions and *J–V* curves of the P3HT:PCBM based BHJ solar cells.

References

1. T.-A. Chen, X. Wu and R. D. Rieke, *J. Am. Chem. Soc.*, 1995, **117**, 233-244.
2. R. H. Lohwasser and M. Thelakkat, *Macromolecules*, 2011, **44**, 3388-3397.
3. V. Senkovskyy, M. Sommer, R. Tkachov, H. Komber, W. T. S. Huck and A. Kiriy, *Macromolecules*, 2010, **43**, 10157-10161.
4. G. Sarasqueta, K. R. Choudhury, J. Subbiah and F. So, *Adv. Funct. Mater.*, 2011, **21**, 167-171.
